# Beyond Traditional Risk Scores: Artificial Intelligence in Coronary Plaque Characterization and Personalized Atherosclerosis Management

**DOI:** 10.3390/jcdd13090469

**Published:** 2026-09-16

**Authors:** Rassulbek Aipov, Timur Saliev, Baurzhan Aipov, Zulfiya Kachiyeva, Aksoltan Oradova, Ildar Fakhradiyev

**Affiliations:** 1 School of Pediatrics, S.D. Asfendiyarov Kazakh National Medical University, Tole Bi Street 94, Almaty 050000, Kazakhstan; 2 Institute for Fundamental and Applied Medical Research, S.D. Asfendiyarov Kazakh National Medical University, Tole Bi Street 94, Almaty 050000, Kazakhstan; 3 Department of Cardiology, S.D. Asfendiyarov Kazakh National Medical University, Tole Bi Street 94, Almaty 050000, Kazakhstan; 4 College of Medicine, Korea University, Seoul 02841, Republic of Korea

**Keywords:** atherosclerosis, artificial intelligence, machine learning, risk stratification, personalized medicine, cardiovascular disease, imaging analysis, predictive modeling

## Abstract

Atherosclerosis remains a leading global cause of cardiovascular morbidity and mortality, yet its insidious progression and multifaceted etiology, spanning genetic, metabolic, and environmental determinants, often delay clinical recognition until adverse events occur. Traditional risk stratification tools, while foundational in preventive cardiology, are constrained by their reliance on limited variables and static linear assumptions, frequently misclassifying individuals at the extremes of risk. This review critically examines the transformative role of artificial intelligence (AI), particularly machine learning (ML) and deep learning (DL), in redefining atherosclerosis management across three interconnected domains. First, we explore how AI-driven predictive models integrate high-dimensional data, from genomics and imaging to real-time wearable metrics, to achieve superior cardiovascular risk stratification compared with conventional scores. Second, we detail AI’s capacity to automate and enhance plaque characterization through advanced imaging analysis, enabling reproducible quantification of burden, composition, and vulnerability markers that are imperceptible to human readers. Third, we investigate AI-powered clinical decision support systems, digital twins, and reinforcement learning approaches that facilitate dynamic, personalized treatment planning tailored to each patient’s evolving profile. We also critically address the ethical imperatives, algorithmic fairness, data privacy, transparency, and accountability, alongside practical challenges of clinical integration, regulatory validation, and health equity.

## 1. Introduction

Atherosclerosis is a progressive and multifactorial vascular disorder characterized by the build-up of lipid-rich plaques within arterial walls. This process is driven by a complex interplay of genetic predisposition, metabolic abnormalities, chronic inflammation, and environmental and behavioral factors [1]. As plaques accumulate and become unstable, they may rupture or erode, leading to life-threatening events such as myocardial infarction, ischemic stroke, or peripheral arterial disease. Despite extensive advances in cardiovascular diagnostics and therapeutics, atherosclerosis remains the leading cause of morbidity and mortality worldwide, representing a substantial public health and economic burden [2,3].

**Figure 1:**
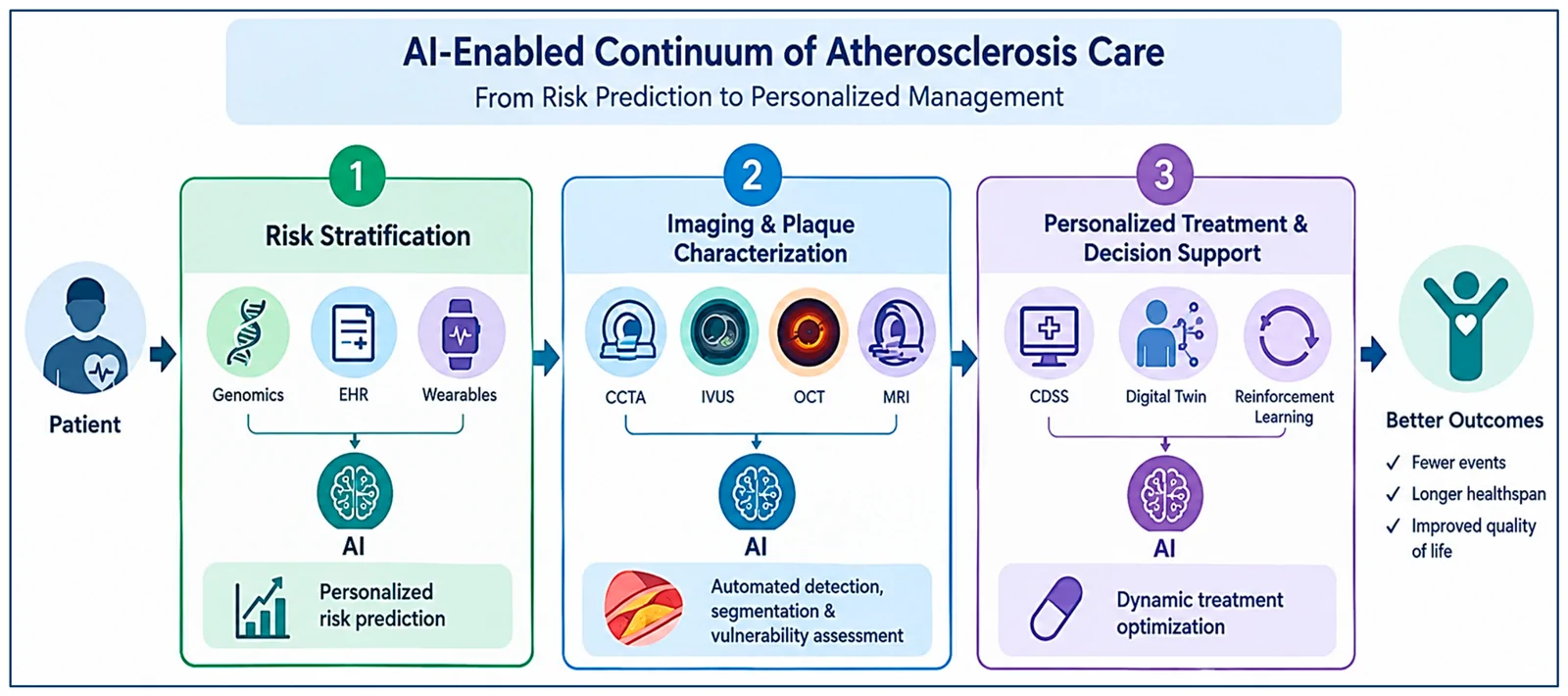
AI-Enabled Continuum of Atherosclerosis Care. This schematic illustrates the three interconnected domains where AI transforms atherosclerosis management: (1) Risk Stratification—integrating multimodal data (EHR, genomics, wearables) for personalized risk prediction; (2) Imaging & Plaque Characterization—automated detection, segmentation, composition analysis, and vulnerability assessment from CCTA, IVUS, OCT, and MRI; (3) Personalized Treatment & Decision Support—AI-powered CDSS, digital twins, and reinforcement learning for dynamic treatment optimization.

One of the central challenges in managing atherosclerosis is the early and accurate identification of individuals at high risk for cardiovascular events. Conventional risk assessment tools, such as the Framingham Risk Score, the ASCVD risk calculator, and the SCORE system, have been foundational in preventive cardiology [4]. However, these models are typically based on a small number of clinical variables and assume static, linear relationships that may not capture the complexity and heterogeneity of disease development across different individuals and populations. Consequently, many patients are either overtreated or undertreated, and subtle high-risk profiles may go undetected until an adverse event occurs (Figure 1).

In this context, artificial intelligence (AI) and its subfields, machine learning (ML) and deep learning (DL), have emerged as transformative tools capable of redefining how atherosclerosis is detected, monitored, and managed [5]. AI systems have demonstrated capability in identifying non-linear patterns, processing high-dimensional datasets, and learning from heterogeneous data sources. Preliminary evidence suggests that AI may generate predictive insights and therapeutic recommendations that are more personalized and adaptive than those generated by traditional models, though prospective validation of clinical benefit remains an ongoing process.

Furthermore, AI enables continuous learning, meaning that prediction models can evolve as new data becomes available [6]. This is particularly relevant in cardiovascular care, where patient conditions and treatment responses often change over time [7,8]. AI-based systems can provide real-time risk assessments, support early interventions, and adjust treatment strategies dynamically, characteristics that are essential for managing a chronic and variable condition like atherosclerosis (Figure 1).

Several recent narrative reviews have discussed the role of AI in cardiovascular disease, including in atherosclerosis (e.g., Wang et al., 2025 [9]). Our work differs by providing a focused synthesis on how AI enhances not only risk prediction but also imaging-based plaque characterization and personalized treatment strategies, while explicitly addressing challenges of clinical translation, ethics, and equity. This targeted scope highlights the practical pathways and barriers toward integrating AI into routine atherosclerosis management.

This review provides a comprehensive examination of the emerging role of AI in atherosclerosis management, with a focus on three core areas: enhanced risk stratification, advanced imaging interpretation and plaque characterization, and personalized treatment and decision support. In addition, we explore the ethical and practical challenges of integrating AI into clinical practice and outline future directions for research and implementation.

### Literature Search Strategy

This narrative review was conducted through a systematic literature search across multiple electronic databases, including PubMed/MEDLINE, Scopus, and Web of Science, covering the period from January 2018 to June 2026. The search strategy combined keywords and MeSH terms related to three core domains: artificial intelligence and machine learning (e.g., “deep learning”, “neural networks”, “machine learning”, “artificial intelligence”), atherosclerosis and cardiovascular disease (e.g., “atherosclerosis”, “coronary artery disease”, “plaque characterization”, “cardiovascular risk”), and clinical applications (e.g., “risk stratification”, “imaging analysis”, “personalized treatment”, “clinical decision support”). Boolean operators were used to refine the search. We included peer-reviewed original research, systematic reviews, meta-analyses, and authoritative narrative reviews that focused on AI/ML techniques for risk prediction, imaging analysis, or treatment personalization in atherosclerosis. Conference abstracts, editorials, and opinion pieces without systematic evidence were excluded. Given the heterogeneity of study designs, a narrative synthesis was performed, organizing findings thematically according to the three core domains: risk stratification, imaging and plaque characterization, and personalized treatment.

## 2. Risk Stratification and Predictive Modeling

As discussed in the Introduction, AI’s capacity to process high-dimensional, multimodal data offers a paradigm shift in cardiovascular risk assessment. Unlike conventional tools, AI models can integrate not only traditional risk factors but also granular data such as genomics, proteomics, metabolomics, imaging biomarkers, socioeconomic status, psychosocial stress, environmental exposures, and longitudinal behavioral data from wearable devices [10,11]. This capacity allows AI systems to build more comprehensive and individualized risk profiles, moving beyond the limitations of traditional scoring systems discussed above.

AI, and specifically ML and DL techniques, offer a potentially transformative approach to cardiovascular risk assessment by enabling the analysis of high-dimensional, multimodal datasets. However, improved predictive discrimination does not automatically translate into improved patient outcomes, and clinical benefit requires prospective validation. Unlike conventional tools, AI models can process vast volumes of structured and unstructured data, identify subtle, non-linear relationships, and uncover hidden patterns that contribute to atherosclerotic risk. These models are capable of integrating not only conventional risk factors but also granular data such as genomics, proteomics, metabolomics, imaging biomarkers, socioeconomic status, psychosocial stress, environmental exposures, and longitudinal behavioral data from wearable devices [12]. This capacity allows AI systems to build more comprehensive and individualized risk profiles.

Recent studies have demonstrated the superiority of AI models in predicting major adverse cardiovascular events (MACE) [13,14]. Algorithms such as random forests, support vector machines (SVMs), gradient boosting machines, and neural networks have shown improved predictive accuracy over traditional models [13,15].

These tools have been trained on large, real-world datasets derived from electronic health records (EHRs), biobanks, and national health databases. Notably, 2023–2025 studies continue to refine AI-based cardiovascular prediction, such as deep learning ECG models for event detection and multimodal risk stratification frameworks that incorporate genetics, imaging, and behavioral data [16,17].

For example, AI algorithms can assimilate laboratory trends, medication adherence patterns, vital signs, and lifestyle data captured from mobile health applications or wearable devices, generating real-time risk alerts [18,19]. Such systems can identify high-risk individuals before clinical deterioration occurs, facilitating earlier interventions and more targeted preventive strategies. In clinical practice, this could mean initiating statins or antihypertensive therapy in patients who would not otherwise meet guideline-based thresholds but are found to have a high AI-predicted short-term risk.

Another key advantage of AI-based risk stratification lies in its adaptive learning capabilities. As more data are accumulated, whether through new patient encounters, changes in clinical practice, or population shifts, AI models can be retrained to reflect contemporary evidence and demographic trends. This flexibility addresses a key limitation of traditional risk calculators, which are static and often become outdated or mis-calibrated as treatment paradigms and population health profiles evolve [20,21].

Moreover, AI can support phenotyping and subgroup discovery, identifying previously unrecognized clusters of patients with shared risk profiles but differing prognoses or therapeutic responses. For instance, AI may reveal a subgroup of young, non-obese patients with elevated inflammatory biomarkers and early arterial stiffness, who are at disproportionately high risk despite appearing low-risk by conventional measures [22,23]. This ability to detect nuanced risk patterns can help clinicians refine diagnostic and treatment strategies beyond the capabilities of standard scoring systems. A comparative review of traditional and AI-based risk stratification in atherosclerosis is provided in Table 1.

In summary, despite these strengths, the clinical adoption of AI for risk stratification is not without challenges. Model transparency and interpretability remain significant hurdles, particularly with complex deep learning architectures, as clinicians may hesitate to trust or act upon “black-box” outputs without clear explanations. To address this, efforts are underway to develop explainable AI (XAI) systems that deliver interpretable, clinician-friendly insights while preserving predictive performance. The broader challenges of validation, interpretability, and clinical integration are discussed further in Evaluating AI Model Performance Section and Section 6.

Several recently developed machine learning-based risk scores illustrate both the promise and the challenges of clinical translation. The ECG-SMART model (Al-Zaiti, 2023) [24] is an ECG-only deep learning tool that estimates individual cardiovascular risk directly from 12-lead tracings, offering a scalable and low-cost approach particularly useful where imaging or laboratory data are unavailable. Its validation in large bio-banks and prospective cohorts underscores its potential, although real-world implementation will require careful threshold selection, integration into ECG workflows, and safeguards for interpreting non-ischemic patterns. Similarly, the PRAISE score [25] was developed in patients with acute coronary syndromes using multicentre datasets, and has shown improved prediction of adverse outcomes beyond conventional risk models. Yet, practical deployment demands re-calibration across diverse hospital settings, ongoing adjustment to account for event prevalence shifts, and clinician-facing explanations of key drivers of risk to build trust.

The SEX-SHOCK score was recently introduced as a machine learning, derived tool to predict the risk of developing cardiogenic shock in patients with acute coronary syndromes (ACS) [26,27,28]. By incorporating sex-specific variables, it demonstrated improved discrimination compared with traditional scores, particularly in highlighting female patients at elevated risk. Importantly, the score is not designed to predict mortality in patients who already have cardiogenic shock, but rather to identify ACS patients at risk of progressing to shock. This early identification is clinically meaningful because it enables the timely initiation of hemodynamic monitoring, escalation of care, or preventive interventions before shock develops. As such, SEX-SHOCK illustrates how AI-based models can refine acute risk stratification through the incorporation of biological and sex-specific differences, complementing established prognostic tools.

Collectively, these examples show how AI-driven scores can refine risk prediction, but also emphasize the operational, technical, and interpretability hurdles that must be overcome for successful clinical uptake. Importantly, the lessons learned from these risk models, particularly regarding calibration, workflow integration, and transparency, are directly relevant to atherosclerosis-focused AI applications, where plaque quantification and vulnerability assessment face similar challenges on the path to routine clinical adoption.

While Section 2 emphasized how AI integrates heterogeneous data to stratify patient-level risk, the next Section 3 focuses on imaging itself, automated plaque detection/segmentation, composition phenotyping, and vulnerability characterization, detailing how image-derived features are generated, validated, and readied for clinical use.

### Evaluating AI Model Performance: Beyond Discrimination

While many AI studies report area under the receiver operating characteristic curve (AUC) as the primary metric of model performance, this measure alone is insufficient to establish clinical utility. Several additional metrics are essential for a comprehensive evaluation.

Calibration refers to the agreement between predicted and observed risks. A model may have high discrimination (AUC) but poor calibration, leading to systematic overestimation or underestimation of risk in specific patient subgroups. Calibration plots and the Hosmer–Lemeshow test are commonly used to assess this aspect.

Decision curve analysis evaluates the net clinical benefit of a model across different risk thresholds, accounting for the relative harms of false-positive and false-negative classifications. This approach is particularly relevant in atherosclerosis management, where the consequences of undertreatment (missed events) and overtreatment (unnecessary medication side effects, costs) must be balanced.

Net reclassification improvement (NRI) and integrated discrimination improvement (IDI) quantify how much better a new model classifies individuals into risk categories compared with a reference model. These metrics are valuable for demonstrating incremental value over traditional risk scores.

External validation in independent, diverse populations is perhaps the most critical step. Many AI models show excellent performance in internal validation but degrade substantially when applied to external cohorts with different demographics, imaging protocols, or disease prevalence. Reporting of external validation should include performance metrics in each validation cohort, with explicit description of population characteristics.

Addressing these methodological gaps is essential for translating promising AI models into tools that can reliably inform clinical decision-making [29].

## 3. Imaging Analysis and Plaque Characterization

Accurate risk stratification is the cornerstone of effective atherosclerosis prevention and management. Traditionally, cardiovascular risk prediction has relied on models such as the Framingham Risk Score, SCORE, and ASCVD risk calculators, which are based on a limited number of clinical parameters, including age, sex, blood pressure, cholesterol levels, smoking status, and diabetes [30,31]. While these tools are widely used and have demonstrated utility in population-level screening, they inherently assume linear and additive relationships among risk factors.

At the same time, imaging technologies such as coronary computed tomography angiography (CCTA), intravascular ultrasound (IVUS), optical coherence tomography (OCT), and magnetic resonance imaging (MRI) have become indispensable tools in assessing atherosclerosis. They provide insights into plaque burden, morphology, composition, and stability, which are critical determinants of future cardiovascular events. Traditionally, however, imaging interpretation has relied heavily on manual evaluation by specialists, a process that is time-consuming, operator-dependent, and subject to variability. AI, particularly DL, offers transformative capabilities for automating and enhancing the accuracy, reproducibility, and prognostic utility of imaging-based plaque assessment. Unlike general risk prediction models that combine multimodal datasets, AI in imaging is uniquely positioned to extract granular plaque features directly from raw imaging data, quantify disease burden, and identify markers of plaque vulnerability with unprecedented precision (Table 2).

AI-based models can handle high-dimensional, multi-source data, including not only traditional clinical variables but also detailed biomarkers, genomic and proteomic data, imaging findings, wearable device outputs, and unstructured data such as clinician notes from electronic health records (EHRs) [32].

### 3.1. Automated Plaque Detection and Segmentation

One of the most impactful contributions of AI in cardiovascular imaging is the automation of plaque detection and segmentation, a task that traditionally required considerable time, expertise, and manual input from radiologists and cardiologists. Manual segmentation is not only labor-intensive but also highly variable, with significant inter- and intra-observer differences, particularly in distinguishing non-calcified from mixed plaques. AI-based algorithms, especially convolutional neural networks (CNNs) and other DL architectures, have demonstrated remarkable accuracy in identifying coronary plaques on coronary computed tomography angiography (CCTA), enabling differentiation between calcified, non-calcified, and mixed plaques with performance metrics comparable to expert readers.

Automated segmentation tools substantially accelerate clinical workflows by rapidly delineating coronary arteries, extracting vessel boundaries, and isolating atherosclerotic lesions across entire datasets within minutes. These tools can automatically generate coronary artery calcium (CAC) scores, quantify total plaque burden, and measure luminal stenosis with high reproducibility. For example, DL-based CCTA platforms can provide per-patient and per-segment analysis of plaque volume and composition, delivering outputs that align closely with intravascular reference standards such as IVUS or OCT. Such automation transforms imaging analysis from a subjective and time-consuming process into a standardized, reproducible, and scalable solution suitable for both clinical practice and large-scale population studies.

Beyond calcium scoring and stenosis quantification, AI-driven segmentation also allows for the identification of high-risk morphological features, such as positive remodeling, spotty calcification, and low-attenuation plaques. By detecting these features reliably, automated systems can contribute not only to descriptive reporting but also to prognostic risk assessment, as these markers are strongly associated with future cardiovascular events. Moreover, integration with radiomics pipelines enables the extraction of hundreds of subtle imaging features (e.g., texture, intensity, and shape), extending the analysis beyond what is visible to the human eye. This offers a more comprehensive phenotyping of plaque biology that may improve the prediction of plaque vulnerability and clinical outcomes. Recent radiomics-based approaches further highlight the ability of AI to link subtle imaging phenotypes with long-term cardiovascular outcomes [33,34,35].

Several commercially available AI platforms have integrated automated plaque segmentation into clinical workflows, providing structured quantitative reports that assist clinicians in therapy optimization, such as intensification of lipid-lowering therapy for patients with high-risk non-calcified plaque burden [36,37]. These tools also hold promise in reducing diagnostic disparities by ensuring consistent plaque assessment across imaging centers with different levels of expertise.

### 3.2. Characterization of Plaque Composition

Beyond mere detection, a critical advancement of AI in atherosclerosis imaging lies in its ability to characterize plaque composition. Coronary plaques are heterogeneous structures that may consist of calcified, fibrous, lipid-rich, or necrotic components, each with distinct biological behavior and prognostic significance. These compositional differences are not merely descriptive: calcified plaques are generally more stable, whereas non-calcified and lipid-rich plaques, particularly those with large necrotic cores, are associated with inflammation, mechanical stress, and an increased risk of rupture. Differentiating these plaque subtypes is therefore essential for assessing patient vulnerability and guiding preventive interventions.

DL models trained on coronary computed tomography angiography (CCTA) and intravascular ultrasound (IVUS) images have achieved high sensitivity and specificity in distinguishing vulnerable, non-calcified plaques from more stable calcified lesions. Automated algorithms can segment coronary plaques into compositional categories, quantify lipid-rich burden, and detect necrotic cores with reproducibility that matches or exceeds expert manual assessment. Such AI-driven quantification is particularly valuable in non-calcified lesions, where manual interpretation is both challenging and prone to inter-observer variability.

Optical coherence tomography (OCT), owing to its near-microscopic resolution, provides unparalleled visualization of plaque microstructure. When combined with AI, OCT can automatically detect thin-cap fibroatheromas (TCFAs), macrophage infiltration, cholesterol crystals, and microchannels, hallmarks of plaque instability that are otherwise time-intensive to evaluate. Automated OCT-based classification pipelines not only accelerate image interpretation but also standardize the detection of these critical markers across centers, which is essential for building robust risk models. Similarly, hybrid models that integrate IVUS or OCT with DL-based feature extraction have shown promise in differentiating necrotic cores and fibrous tissue, further enhancing the granularity of compositional analysis.

Beyond single-modality assessments, AI also enables radiomics-based characterization, in which hundreds of quantitative imaging features, such as pixel intensity, spatial texture, and morphological patterns, are extracted from CCTA datasets. These radiomic signatures provide deeper insights into plaque biology, capturing subtle tissue heterogeneity that is imperceptible to human readers. Early studies have shown that radiomic markers of lipid-rich or inflamed plaques correlate strongly with future acute coronary syndromes, suggesting that AI-driven compositional analysis could serve as an early biomarker of clinical events.

### 3.3. Identification of Plaque Vulnerability

A critical frontier in atherosclerosis management is the ability to identify plaques that are prone to rupture and precipitate acute coronary syndromes (ACS). While luminal stenosis has historically been used as the principal marker of disease severity, it is now clear that many myocardial infarctions arise from non-obstructive but biologically “vulnerable” plaques. These lesions are typically characterized by a thin fibrous cap, large lipid or necrotic core, active inflammation, and positive vascular remodeling. Detecting such features non-invasively and at scale is one of the most promising contributions of artificial intelligence to cardiovascular imaging.

AI algorithms applied to coronary computed tomography angiography (CCTA) have demonstrated the ability to detect imaging features of plaque vulnerability, including low-attenuation plaque (<30 Hounsfield units), positive remodeling, spotty calcification, and the napkin-ring sign. These high-risk features have been validated in multiple longitudinal cohorts as predictors of future adverse cardiovascular events. Automated AI pipelines not only accelerate the identification of these markers but also provide consistent quantification across large datasets, addressing a major limitation of manual interpretation where inter-observer variability has historically been substantial.

Radiomics, an emerging AI-driven field, significantly enhances vulnerability assessment by extracting hundreds to thousands of quantitative imaging features, such as shape descriptors, texture heterogeneity, intensity gradients, and higher-order statistical patterns, that are imperceptible to the human eye. These radiomic signatures can capture subtle biological processes like lipid infiltration or microcalcification, which underlie plaque instability. Early clinical studies have shown that radiomic profiles derived from CCTA are independently associated with major adverse cardiovascular events, even after adjustment for conventional risk factors and traditional imaging markers. Such data highlight the potential of radiomics to refine risk prediction models and uncover latent patterns that would otherwise remain hidden.

The ability of AI to characterize vulnerability at the plaque level rather than solely at the patient level represents a paradigm shift in risk assessment. Instead of considering a patient’s overall risk as uniform across all vascular segments, AI can assign risk scores to individual plaques, highlighting those most likely to rupture. This granularity may inform precision interventions, such as targeted intensification of lipid-lowering or anti-inflammatory therapy, closer surveillance, or in selected cases, preventive revascularization. Such lesion-specific risk assessment aligns with the emerging concept of precision cardiology, where therapy is tailored not only to the patient but also to the biology of specific lesions.

#### Synthesis of Available Studies on AI-Assisted CT Plaque Analysis

A growing body of literature has evaluated AI-assisted CCTA for plaque characterization across diverse cohorts and clinical settings. Klüner et al. (2024) provided a comprehensive review highlighting AI applications in detecting obstructive plaques, assessing plaque volumes and vulnerability, monitoring plaque progression, and providing risk assessment [38].

Several large-scale validation studies have demonstrated the clinical utility of AI-based plaque quantification. A consensus statement from the QCI Study Group (2025) established that AI-supported analysis of coronary atherosclerosis on CCTA allows age-adjusted and gender-adjusted percentile curves to be generated for atherosclerosis imaging biomarkers [39]. In a large Chinese cohort study, fully automated AI coronary plaque analysis systems were used to reveal age- and sex-specific distribution charts for atherosclerotic plaque characteristics, employing coronary segmentation and labeling models, plaque detection and segmentation models, and models for classifying high-risk plaque features [40].

AI-enabled plaque characterization has also been applied to specific patient populations. In a study of 117 women with nonobstructive atherosclerosis, AI-based software quantitatively analyzed non-calcified plaque, low-attenuation plaque, and calcified plaque volumes and burdens, establishing a basis for understanding angina in this population [41]. Recent studies have shown that deep learning networks can be applied for rapid automated segmentation of coronary plaque from CCTA, with AI-enabled measurement of total plaque volume predicting future heart attack.

A roadmap on the use of AI for imaging of vulnerable atherosclerotic plaque in coronary arteries, developed by an interdisciplinary group of experts, provides consensus recommendations on AI applications in both non-invasive and invasive coronary imaging. Similarly, Xia et al. (2024) reviewed enhancing coronary artery plaque analysis via AI-driven cardiovascular CT, highlighting both technical advances and remaining challenges [42].

Despite these advances, key evidence gaps remain. Barriers to widespread clinical adoption include data heterogeneity, algorithmic bias, limited model transparency, insufficient prospective validation, regulatory challenges, and incomplete integration into clinical workflows. Many identified models are not currently suitable for clinical use, primarily due to methodological flaws and underlying biases. The field would benefit from standardized evaluation metrics, larger prospective validation studies, and clearer regulatory pathways.

### 3.4. Integration of Imaging with Clinical Outcomes

AI-driven imaging analysis does more than describe arterial morphology; it bridges the gap between anatomical observations and clinical prognosis by integrating imaging-derived features with outcomes data to build predictive models. For example, CCTA-based AI models that quantify high-risk plaque features, such as low-attenuation plaque, positive remodeling, and overall plaque burden, have been validated in prospective registries and clinical trial datasets, consistently demonstrating predictive value for myocardial infarction and other adverse cardiovascular events beyond traditional risk scores. Importantly, these models have shown incremental benefit even in statin-treated populations, highlighting their utility in addressing residual cardiovascular risk.

Similarly, AI applications in IVUS and OCT extend beyond static characterization of plaque composition. By analyzing peri-stent neointimal growth, vascular healing patterns, and microstructural changes, AI algorithms can predict long-term complications such as stent restenosis or late stent thrombosis. These prognostic insights are particularly valuable in tailoring post-intervention therapy, optimizing antiplatelet regimens, and informing follow-up imaging strategies.

By linking imaging phenotypes to hard outcomes, AI enables cardiovascular imaging to evolve from a primarily diagnostic modality into a powerful prognostic and decision-support tool. This shift allows clinicians not only to visualize existing disease but also to anticipate its clinical trajectory, thereby enabling earlier and more targeted preventive interventions.

### 3.5. Standardization and Reproducibility

One of the enduring challenges in plaque imaging has been the high degree of inter-observer variability and the lack of reproducibility across imaging modalities, institutions, and even within the same reader over time. AI helps overcome these limitations by providing standardized, automated workflows that generate objective, quantitative measurements of plaque burden, composition, and morphology. For example, automated quantification of coronary artery calcium (CAC) scoring has demonstrated near-perfect agreement with manual scoring while significantly reducing processing time and human resource demands. This not only streamlines clinical workflows but also enhances consistency in patient risk assessment.

Similarly, AI-based IVUS and OCT interpretation pipelines allow for the reliable and repeatable identification of fibrous caps, necrotic cores, calcification, and other microstructural features across large datasets. This consistency is crucial in multicentre studies, where variability in manual interpretation has historically limited the generalizability of findings. By reducing subjectivity, AI facilitates broader research collaborations, enables meta-analyses across diverse populations, and supports regulatory validation of novel imaging biomarkers.

A critical consideration for the clinical deployment of AI-based imaging systems is the variability introduced by different scanner vendors, acquisition protocols, and preprocessing pipelines. Proprietary image reconstruction algorithms can introduce systematic biases that affect model performance. Current efforts in image harmonization, domain adaptation, and federated model training are addressing this issue by enabling AI systems to learn from diverse datasets without centralizing sensitive data. Establishing standards for data normalization and inter-institutional calibration will be essential to ensure reproducibility, regulatory compliance, and robust generalizability of AI solutions across healthcare networks.

In addition, standardized AI-driven outputs create the foundation for building large-scale imaging biobanks, which can be leveraged for training next-generation predictive models. Such reproducibility also accelerates the translation of imaging biomarkers into clinical practice and strengthens confidence in using AI-derived plaque metrics for longitudinal patient monitoring and therapeutic decision-making.

### 3.6. Real-World Applications and Emerging Trends

In clinical practice, AI-based imaging tools are beginning to show tangible benefits. Automated CCTA platforms are being used to triage patients for invasive angiography by quantifying both luminal stenosis and high-risk plaque features. In advanced centers, AI tools have been integrated into hybrid decision-support systems that combine imaging-derived plaque characterization with clinical variables to optimize therapy, such as selecting patients for intensified lipid-lowering or anti-inflammatory treatment. Emerging directions include the creation of imaging-based “digital twins” of the coronary vasculature, enabling virtual simulation of disease progression and therapeutic interventions at the individual patient level.

AI-driven models are particularly advantageous in their ability to incorporate time-varying data, enabling dynamic risk prediction rather than relying on static, one-time assessments. For example, by continuously integrating updated laboratory values, medication adherence data, and blood pressure readings from home monitors, AI tools can provide real-time risk estimates and alert clinicians to early signs of disease progression or instability [43,44]. By leveraging vast datasets, AI can identify distinct subgroups or phenotypes within populations that share similar risk factor patterns but differ in disease progression or treatment response. For example, some AI models have uncovered unique risk profiles among younger patients or those with atypical lipid panels, who might otherwise be overlooked by traditional algorithms [45,46].

As discussed in Evaluating AI Model Performance Section, robust external validation and interpretability remain essential for clinical translation [47]. Techniques such as SHAP (Shapley Additive Explanations) and LIME (Local Interpretable Model-agnostic Explanations) are being increasingly applied to make AI predictions more understandable to clinicians and patients. By narrowing its focus to imaging, this section emphasizes how AI transforms plaque analysis beyond general risk stratification. From automated plaque detection and composition analysis to radiomics-driven vulnerability assessment, AI enables a more reproducible, precise, and prognostically meaningful use of imaging data. This shift not only enhances diagnostic accuracy but also supports precision cardiology by linking imaging-derived features directly to patient outcomes and guiding personalized interventions.

Beyond algorithmic accuracy, the successful translation of AI into cardiovascular diagnostics depends on demonstrable value at the health system level. Cost-effectiveness analyses should evaluate not only diagnostic yield but also workflow efficiency, resource utilization, and long-term outcomes such as reduced hospitalisations or unnecessary testing. Integrating AI-assisted tools into existing reimbursement frameworks requires evidence that automation can lower costs while maintaining or improving clinical quality. Scalable implementation strategies, such as cloud-based deployment, interoperability with electronic health records, and integration into standardized imaging workflows, are key to achieving payer acceptance and widespread adoption across diverse healthcare settings.

### 3.7. Clinical Translation and Implementation of AI Imaging Tools

Real-world deployment of AI imaging tools requires more than high diagnostic accuracy; it also depends on predictable integration into clinical workflows, robust governance structures, and sustainable reimbursement models. Without these elements, even the most accurate algorithms risk remaining confined to research settings rather than achieving widespread clinical adoption.

Workflow integration. For AI tools to be useful, their outputs—such as automated calcium scoring, plaque quantification, or vulnerability phenotyping—must appear directly within the clinician’s existing workflow. This is increasingly facilitated by open interoperability standards. SMART on FHIR enables secure app launch and data exchange across different systems, while HL7 CDS Hooks allow AI-driven decision support to be triggered at key clinical moments (e.g., patient review or order entry). By embedding outputs seamlessly into the electronic health record (EHR), these standards reduce “swivel-chair” burden, promote vendor-agnostic deployment, and allow more sophisticated applications to be launched when deeper analysis is needed.

Regulatory pathways. Successful translation also requires clear regulatory oversight. In the United States, AI imaging tools that guide diagnosis or management are regulated as Software as a Medical Device (SaMD). The FDA’s Good Machine Learning Practice (GMLP) framework emphasizes data quality, model transparency, human oversight, ongoing post-market monitoring, and the agency is exploring “Predetermined Change Control Plans” to accommodate adaptive learning algorithms. In Europe, the AI Act classifies most medical AI applications as “high-risk,” requiring compliance with risk management, data governance, transparency, and human oversight, in addition to MDR/IVDR requirements. The UK MHRA is similarly advancing its Software and AI as a Medical Device change program, clarifying expectations across the entire lifecycle.

Reimbursement. Another determinant of clinical adoption is reimbursement. A notable precedent is the non-invasive FFRCT analysis, which achieved Category I CPT coding and broad CMS coverage, setting a pathway for other AI-enabled cardiovascular imaging solutions. More recently, several Medicare Administrative Contractors issued coverage for AI-based CCTA plaque analysis in late 2024, and the AMA has announced Category I coding for additional AI plaque quantification services with implementation dates extending into 2026. These developments demonstrate that once an AI tool is validated, standardized, and integrated into workflow, reimbursement can follow, paving the way for routine clinical use. The operational and technical challenges of achieving this integration, including workflow integration and regulatory validation, are discussed further in Section 6.

Operationalization and monitoring. Finally, clinical implementation requires governance beyond deployment. Institutions must define intended-use populations, continuously monitor data drift and calibration, and track concordance between human and AI outputs as well as their impact on clinical decision-making. Embedding usage analytics and conducting periodic site-specific revalidation are essential for maintaining performance across different scanners, imaging protocols, and patient demographics. Such operational safeguards ensure reliability, foster clinician trust, and help meet regulatory and medico-legal requirements.

### 3.8. Clinical Readiness of AI Imaging Tools: Established Versus Emerging Applications

It is important to distinguish between AI imaging tools that have achieved regulatory approval and clinical implementation and those that remain at the research or early-validation stage. Among the most mature applications is automated coronary artery calcium (CAC) scoring, which is now embedded in many commercial CT workstations and has been validated in large populations. AI-based CCTA plaque quantification and FFRCT analysis have received FDA clearance and are increasingly used in clinical practice, with established CPT coding and reimbursement pathways.

In contrast, radiomics-based vulnerability assessment, despite promising retrospective data, remains primarily investigational. Similarly, the use of AI for predicting plaque progression or for guiding anti-inflammatory therapy selection has not yet been prospectively validated in large-scale trials. Table 3 provides a classification of AI imaging tools according to their current clinical readiness.

## 4. Personalized Treatment and Decision Support

The management of atherosclerosis is evolving rapidly with the emergence of personalized medicine, an approach that seeks to tailor therapeutic strategies to the unique characteristics of each patient. The traditional “one-size-fits-all” model is increasingly recognized as inadequate for addressing the complex and heterogeneous nature of atherosclerotic disease. Individual variability in genetic background, biomarker expression, comorbid conditions, lifestyle, and disease progression necessitates a more nuanced, data-driven approach. AI, particularly through ML and DL, has emerged as a powerful enabler of personalized cardiovascular care, capable of integrating vast and diverse data sources to guide more precise, effective, and individualized treatment decisions.

AI algorithms can analyze and synthesize high-dimensional data sets, including electronic health records (EHRs), genomic and proteomic data, metabolomics, advanced imaging findings, and even real-time information from wearable devices. By uncovering hidden patterns and modeling complex interactions between variables, AI can predict disease trajectories, stratify risk more accurately, and estimate individual responses to specific treatments [48]. This capacity forms the foundation for personalized treatment planning in atherosclerosis, moving beyond population averages to patient-specific therapeutic insights (Figure 1). Emerging 2024–2025 studies underscore the potential of digital twins and reinforcement learning to enable dynamic, individualized treatment strategies, reinforcing the trend toward precision cardiology [49,50,51,52].

One of the most impactful applications of AI is the development of clinical decision support systems (CDSS), which assist healthcare providers by delivering evidence-based, personalized recommendations at the point of care [53]. In atherosclerosis management, AI-powered CDSS can support clinicians in selecting optimal lipid-lowering therapies based on a patient’s baseline lipid profile, genetic polymorphisms, and previous treatment responses [54] (Figure 1). These systems can also identify patients who may benefit from novel therapeutic agents such as PCSK9 inhibitors, bempedoic acid, or anti-inflammatory medications, and help tailor drug combinations to maximize efficacy and minimize side effects [55]. Beyond pharmacologic decisions, AI tools can recommend individualized lifestyle modification plans, adjusting dietary and exercise advice based on personal preferences, behavioral tendencies, and readiness for change.

Moreover, AI can model and predict adverse effects of treatment using data from large patient populations, enabling risk mitigation strategies tailored to the individual [8]. Predictive analytics may highlight, for example, patients with renal impairment who are at higher risk for statin-associated side effects or identify those with poor medication adherence patterns, prompting alternative therapeutic approaches [56,57]. Wang et al., in their review “Machine learning in cardiovascular risk assessment: Towards a precision medicine approach” (Wang et al., 2025) [9] highlight limitations of traditional risk scores and argue that ML can integrate diverse data (clinical, ECG, imaging, omics) to enable more precise, individualized prediction. Strengths include its broad scope, balanced discussion of opportunities and challenges, and emphasis on prevention and therapeutic targeting. It could be strengthened by more concrete case examples, validation data, and attention to ethics and equity. Overall, it presents a timely vision of ML as a driver of precision cardiology, while noting key hurdles in translation to clinical practice.

A particularly promising innovation is the use of reinforcement learning, an AI technique that mimics human learning through trial and error, to continuously optimize treatment over time. Unlike traditional models that offer static recommendations, reinforcement learning can adapt to a patient’s evolving condition and real-time health data, adjusting interventions dynamically to achieve the best possible long-term outcomes [58]. This is especially valuable in chronic disease management, where continuous fine-tuning of treatment based on feedback (such as blood pressure readings, lipid levels, or physical activity data) can significantly improve control and prognosis.

Another emerging concept is the creation of digital twins, virtual replicas of patients built using AI models that simulate physiological responses to various interventions. These digital twins can be used to test and compare multiple treatment options in a simulated environment before applying them in clinical practice, thereby supporting truly precision-based decision-making [59,60]. By predicting how a particular intervention might affect a specific patient’s cardiovascular system, digital twins offer a powerful new tool for personalized care planning (Figure 1).

**Figure 2:**
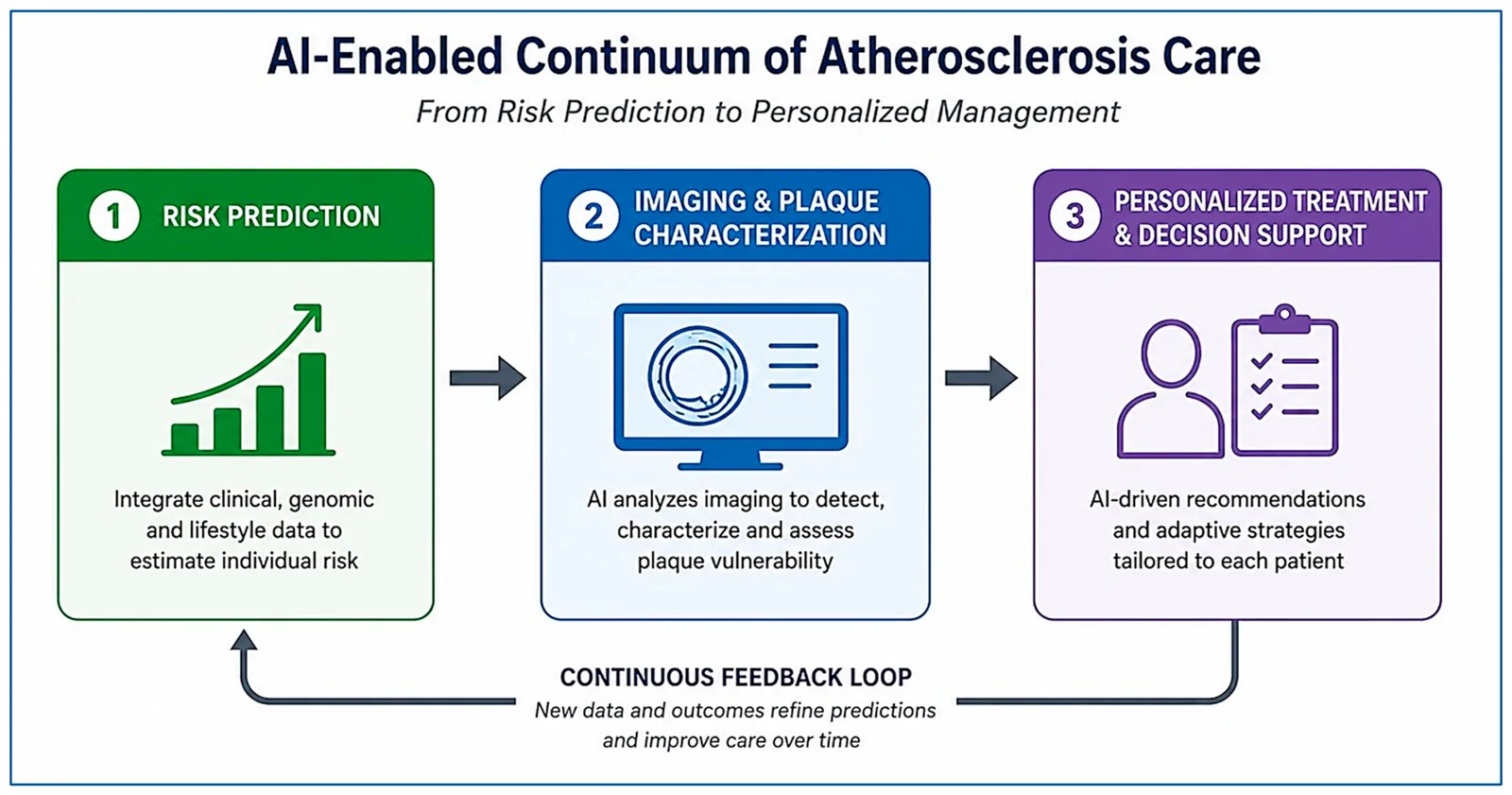
AI-Enabled Continuum of Atherosclerosis Care. The schematic illustrates three interconnected domains in which artificial intelligence (AI) can support personalized atherosclerosis management: (1) Risk Prediction, integrating clinical, genomic, and lifestyle data to estimate individual cardiovascular risk; (2) Imaging and Plaque Characterization, using AI-based analysis to detect, characterize, and assess plaque vulnerability; and (3) Personalized Treatment and Decision Support, providing individualized recommendations and adaptive management strategies.

Furthermore, AI has the potential to enhance shared decision-making by translating complex risk assessments and treatment outcomes into understandable, patient-friendly formats. By providing patients with personalized insights into the risks and benefits of various options, AI can support more informed and participatory healthcare, improving patient engagement, adherence, and satisfaction (Figure 2).

Despite the promise of AI in personalized treatment, several challenges remain. Ensuring model transparency and interpretability is critical to gain clinician trust and meet regulatory requirements. Models must be auditable, explainable, and integrated seamlessly into existing clinical workflows. In addition, data privacy and security must be rigorously maintained, especially when working with sensitive genetic or biometric information. Addressing algorithmic bias through diverse and representative training datasets is also essential to prevent disparities in care and ensure equitable application across different populations.

It is important to emphasize that AI-guided treatment recommendations should currently be considered decision-support tools rather than autonomous treatment decision-makers. While AI can provide evidence-based suggestions for lipid-lowering therapy selection, anti-inflammatory treatment, or lifestyle modification, the final treatment decision must remain with the clinician, who integrates AI recommendations with patient preferences, clinical context, and other factors not captured in the model. Prospective evidence demonstrating that AI-guided treatment recommendations improve patient outcomes beyond standard care remains limited, and AI should be used as an adjunct to, not a replacement for, clinical judgment.

In conclusion, artificial intelligence is poised to revolutionize the personalized treatment of atherosclerosis by enabling the integration of multidimensional patient data into actionable, individualized care strategies. From intelligent decision support and dynamic treatment optimization to digital twin simulation and enhanced patient communication, AI holds immense potential to improve outcomes and transform cardiovascular care. Continued research, interdisciplinary collaboration, and responsible implementation will be vital to fully realize the benefits of AI in personalizing atherosclerosis management.

## 5. Ethical Issues for Artificial Intelligence in Atherosclerosis

The integration of AI into the diagnosis, risk stratification, and management of atherosclerosis offers transformative potential. However, alongside its technical and clinical benefits, the application of AI raises a series of important ethical concerns that must be proactively addressed to ensure its responsible and equitable use. These ethical issues span patient autonomy, algorithmic fairness, data privacy, accountability, and the evolving role of clinicians in AI-assisted care.

One of the most pressing ethical concerns is algorithmic bias. AI systems are only as objective as the data on which they are trained (Table 4). If training datasets are skewed, underrepresenting certain ethnicities, genders, age groups, or socioeconomic backgrounds, AI models may generate biased predictions that systematically disadvantage specific populations [61,62]. For example, recent evaluations of cardiovascular AI models have demonstrated differences in predictive performance according to race, sex, and age. An electronic health record-based study of 109,490 patients found that machine-learning models predicting 10-year coronary heart disease, myocardial infarction, and stroke risk showed lower true-positive rates and a tendency to underestimate risk in women compared with men. Similarly, several cardiovascular prediction models have demonstrated poorer discrimination in Black patients than in White patients, potentially resulting in less accurate identification of individuals who would benefit from preventive interventions [63].

Population bias can also directly affect treatment classification. A 2024 analysis of a national cardiovascular risk prediction tool found that changing the race-specific equation altered atherosclerotic cardiovascular disease (ASCVD) risk classification in approximately 17% of individuals, while use of the alternative equation would have resulted in statin recommendations changing for approximately 10% of black patients [64]. These findings illustrate how apparently small differences in algorithm design can translate into clinically meaningful differences in preventive treatment.

Bias may also arise from the technologies used to generate the data themselves. For example, systematic reviews have demonstrated that pulse oximetry can produce different oxygen-saturation measurements according to skin pigmentation, illustrating how apparently objective physiological measurements can contain population-dependent measurement error that may subsequently be incorporated into AI models [65]. In atherosclerosis, analogous biases could occur when AI systems are trained predominantly on imaging, ECG, genomic, or EHR data from particular demographic populations and subsequently applied to underrepresented groups. This could lead to underdiagnosis, inaccurate risk stratification, or inappropriate treatment recommendations for patients whose characteristics are poorly represented in the training data.

Ethical AI development therefore requires deliberate efforts to ensure that training and validation datasets are diverse and representative, together with systematic evaluation of model performance across age, sex, ethnicity, socioeconomic status, and other clinically relevant subgroups. Importantly, simply removing demographic variables does not necessarily eliminate bias, because AI models can learn indirect proxies for these characteristics from clinical and socioeconomic data [63].

AI systems are fundamentally dependent on the datasets used for their training and validation; if these datasets lack sufficient diversity or are skewed toward certain demographics, the resulting models may inadvertently perpetuate or even amplify existing health disparities. This is especially critical for marginalized and underserved populations, including various ethnic minorities, lower socioeconomic groups, women, and elderly patients, who are historically underrepresented in clinical datasets. Such bias can lead to inaccurate risk predictions, misdiagnoses, or suboptimal treatment recommendations for these vulnerable groups, thereby widening inequities in cardiovascular outcomes.

Ensuring equitable access to AI-enabled cardiovascular care is equally important. The introduction of advanced AI tools should not be limited to well-resourced healthcare institutions or regions with sophisticated infrastructure, as this may exacerbate disparities between urban and rural areas or between high- and low-income countries. Strategies to achieve equity include designing low-cost, scalable AI solutions, promoting inclusive clinical validation that spans diverse populations, and investing in digital infrastructure and training to support broad adoption.

Patient data governance presents additional ethical and practical challenges. AI development and deployment rely on vast quantities of personal health information, which may include sensitive clinical records, imaging data, genomics, and continuously streaming data from wearable devices. Protecting patient privacy requires adherence to stringent legal frameworks such as the General Data Protection Regulation (GDPR) in Europe and the Health Insurance Portability and Accountability Act (HIPAA) in the United States. These regulations mandate secure data storage, controlled access, transparency concerning data usage, and mechanisms for obtaining informed consent specifically covering AI-related data processing activities. Furthermore, patients should retain autonomy over their data, with options to opt in or out, where feasible, without compromising their standard clinical care.

To address such complex ethical issues, several emerging international guidelines offer comprehensive frameworks. The World Health Organization’s (WHO) “Ethics and Governance of Artificial Intelligence for Health” provides principles emphasizing accountability, transparency, inclusivity, and respect for human rights to guide AI integration in health settings [66,67]. The European Commission’s proposed Artificial Intelligence Act classifies medical AI technologies as “high-risk” and imposes mandatory requirements for transparency, risk management, and human oversight. In the United States, the Food and Drug Administration’s (FDA) Good Machine Learning Practice (GMLP) framework focuses on data quality, model validation, transparency, and continuous monitoring over the AI lifecycle to ensure safety and effectiveness.

From a clinical safety perspective, minimizing false negatives is paramount, particularly in moderate-to-high-risk individuals where delayed intervention can significantly increase the likelihood of major adverse cardiac events (MACE). Therefore, evaluation of AI-based diagnostic systems should emphasize not only overall accuracy but also sensitivity thresholds and uncertainty quantification. Risk-averse classification strategies, such as confidence calibration, ensemble modeling, and human-in-the-loop verification, can help mitigate false negatives. Prospective validation in real-world clinical environments is essential to ensure that AI systems maintain safety margins that are comparable to or exceed current diagnostic standards.

Another core issue is data privacy and informed consent. AI models in atherosclerosis care rely on large volumes of patient data, often aggregated from electronic health records, imaging studies, genomics, and wearable devices (Table 4). The ethical use of this data necessitates strict adherence to privacy regulations and ethical norms, including transparency about how patient information is collected, stored, and used [68,69]. Patients should be informed when their data is being used to train or operate AI systems, and, where possible, should be given the option to opt out without compromising their standard of care. Moreover, as AI systems increasingly rely on real-time data streams from personal health technologies, new frameworks are needed to govern consent, ownership, and use of continuously collected health information.

Transparency and explainability represent another significant ethical challenge (Table 4). Many AI models, especially deep learning systems, are inherently opaque, making it difficult for clinicians and patients to understand how a particular decision or prediction was reached [62]. This “black-box” nature of AI undermines trust and limits the ability of users to question or contest machine-generated recommendations. In the context of life-altering decisions, such as initiating invasive cardiovascular procedures or selecting high-cost therapies, explainability becomes not only a technical goal but also an ethical imperative. The development of explainable AI (XAI) systems is crucial to uphold the principles of informed decision-making and shared clinical responsibility [70].

Accountability and liability further complicate the ethical landscape. As AI tools begin to play more central roles in clinical decision-making, questions arise about who is responsible when an AI-generated recommendation leads to harm [71]. Is it the clinician who accepted the suggestion, the institution that deployed the system, or the developers of the algorithm? Clear frameworks are needed to define accountability, especially in scenarios where AI errors result from flawed data, programming issues, or unanticipated clinical contexts [72]. Establishing legal and professional standards for the oversight and safe use of AI is essential to protect patients and guide clinicians.

There are also ethical concerns regarding the dehumanization of care. As AI tools become more prevalent, there is a risk that clinical interactions may become overly data-driven, reducing opportunities for empathy, individualized judgment, and the therapeutic relationship between doctor and patient [73]. While AI can offer precision, it cannot replicate the nuance of human communication, particularly when discussing sensitive diagnoses or navigating complex treatment choices. Ethical implementation should therefore emphasize the role of AI as a supportive tool that augments, rather than replaces, human judgment and compassion in clinical care.

Moreover, access to AI technologies raises questions of justice and equity. Advanced AI tools may be expensive to develop, maintain, and integrate into healthcare systems. If access is limited to well-resourced hospitals or wealthier nations, disparities in cardiovascular outcomes could widen between different regions and socioeconomic groups [12,74].

In conclusion, while artificial intelligence holds great promise for advancing the prevention, diagnosis, and treatment of atherosclerosis, its ethical implementation demands careful attention to issues of fairness, privacy, transparency, accountability, and human-centered care (Figure 2). Ethical frameworks must evolve in parallel with technological innovation to ensure that AI serves as a tool for enhancing, not compromising, equity, trust, and the moral foundations of medical practice. The responsible development and governance of AI in cardiovascular medicine will be critical to realizing its full potential for societal benefit.

## 6. Challenges and Future Directions

Despite its transformative potential, the application of AI in the management of atherosclerosis is not without substantial challenges (Figure 2). These limitations span technical, ethical, clinical, and organizational domains and must be carefully addressed to ensure safe, effective, and equitable integration of AI technologies into routine cardiovascular care.

One of the foremost concerns is data privacy and security. AI models rely on access to vast amounts of sensitive patient data, including electronic health records (EHRs), imaging files, genomic sequences, and data from wearable devices. Ensuring the confidentiality and protection of this information is a fundamental requirement [75]. Regulatory compliance with data protection laws such as the General Data Protection Regulation (GDPR) in Europe or the Health Insurance Portability and Accountability Act (HIPAA) in the U.S. must be rigorously maintained [76,77,78]. Moreover, new approaches such as federated learning, where AI models are trained across decentralized data sources without sharing raw data, are emerging as promising strategies to mitigate privacy risks while still enabling model development (Figure 2).

Another critical barrier is the need for high-quality, annotated, and representative datasets (Figure 2). The success of machine learning algorithms depends heavily on the quality of the data they are trained on. Inconsistent data entry, missing values, and varying standards across institutions can impair model performance and generalizability [79]. Furthermore, datasets must adequately reflect the diversity of the patient population in terms of ethnicity, age, sex, comorbidities, and socioeconomic background to avoid algorithmic bias. Models trained on homogeneous or skewed datasets may produce biased predictions that could perpetuate health disparities. Addressing these issues requires deliberate efforts to collect, curate, and standardize multi-source data across different healthcare settings, particularly from underrepresented populations. The importance of data diversity, representativeness, and the transparency of AI outputs is a recurring theme throughout this review, particularly in Section 5 on ethics.

Equally important is the challenge of model interpretability and transparency. Most AI algorithms, especially deep learning models, are considered “black boxes,” meaning their internal logic is not readily understandable to clinicians (Figure 2). For AI to be accepted and trusted in clinical decision-making, it must provide explainable outputs that can be interpreted and justified in the context of patient care. This has led to the development of explainable AI (XAI) techniques, such as Shapley Additive Explanations (SHAP) and Local Interpretable Model-Agnostic Explanations (LIME), which aim to reveal how input features contribute to a given prediction [80]. However, these tools are still evolving, and translating their outputs into clinically meaningful guidance remains a work in progress.

Regulatory and legal frameworks also lag behind the pace of AI innovation. At present, few AI-based tools have received full regulatory approval for widespread clinical use in cardiovascular medicine. Regulatory agencies are still developing methodologies for validating and certifying AI algorithms, especially those that are adaptive and continuously learning [81]. The establishment of robust and transparent clinical trial frameworks for AI tools, comparable to those used for pharmaceuticals, is necessary to ensure efficacy, safety, and reproducibility.

Integration into existing healthcare workflows presents another major challenge. Many AI systems operate in isolation from hospital information systems or require additional manual steps, which can disrupt clinical routines and reduce adoption [82]. To be useful in practice, AI tools must be embedded seamlessly into electronic medical records and clinical decision pathways, delivering insights at the right time and in a user-friendly format [83]. Interoperability, user interface design, and implementation science principles are therefore essential to operational success. This challenge of transparency, along with the related issue of algorithmic bias, was introduced in Section 2 in the context of risk stratification and is explored in detail in Section 5.

An often-overlooked barrier is the lack of AI literacy among healthcare professionals. For clinicians to effectively use AI tools, they must understand the basic principles of algorithm development, performance evaluation, and limitations [84,85]. Integrating AI education into medical curricula and offering continuous professional development programs is crucial for building the confidence and competence needed for real-world application.

Looking ahead, the future development of AI in atherosclerosis care should be guided by a few strategic imperatives. First, multi-center and international collaborations are essential to create large, diverse, and well-annotated datasets that can support the development of generalizable and unbiased models [86]. Second, cross-disciplinary cooperation among cardiologists, data scientists, engineers, ethicists, and policymakers must be fostered to ensure that AI tools are clinically relevant, ethically sound, and socially responsible [87]. Third, there is a pressing need for standardized evaluation metrics and benchmarking systems to assess the performance of AI tools across different populations and clinical scenarios.

In conclusion, while AI holds enormous promise for transforming the diagnosis, risk prediction, and personalized management of atherosclerosis, realizing this potential requires addressing significant challenges related to data quality, fairness, explainability, regulation, and clinical integration. Through strategic investment, interdisciplinary collaboration, and thoughtful governance, the field can move towards an AI-enabled future in which cardiovascular care is more accurate, equitable, and individualized.

## 7. Review Limitations

This review has several limitations that should be acknowledged. First, as a narrative review, it is subject to potential selection bias in the literature cited, despite our systematic search strategy. Second, the rapid pace of AI innovation means that some findings may become outdated quickly; we have attempted to prioritize recent publications (2018–2026) but cannot guarantee comprehensiveness. Third, the heterogeneity of AI methodologies, datasets, and validation approaches across studies makes direct comparison of model performance difficult. Fourth, we have focused primarily on English-language publications, which may exclude relevant work from non-English sources. Fifth, the evidence base for many AI applications remains preliminary, and our classification of clinical readiness reflects current understanding but may change as new evidence emerges. Finally, we have not performed a formal meta-analysis or quality assessment of individual studies, which limits the ability to quantify effect sizes or compare models rigorously. Despite these limitations, we believe this review provides a balanced and comprehensive overview of the current state and future directions of AI in atherosclerosis management.

## 8. Conclusions

AI holds considerable promise to advance the understanding and management of atherosclerosis. By moving beyond the constraints of traditional models, AI may enable more accurate risk prediction, more consistent interpretation of imaging data, and more personalized treatment strategies. Its ability to analyze diverse data inputs, including genomics, imaging, electronic health records, and real-time sensor data, positions AI as a potentially transformative tool in cardiovascular medicine.

However, realizing this potential requires addressing significant challenges. The current evidence base for many AI applications remains preliminary, with limited prospective validation and few demonstrations of improved patient outcomes. Model transparency, regulatory approval, data privacy, and algorithmic bias must be carefully managed. AI tools should be viewed as decision-support systems that augment, rather than replace, clinical judgment.

Looking forward, the successful integration of AI into atherosclerosis care will depend on: (1) rigorous prospective validation of clinical utility and safety; (2) development of transparent, interpretable models; (3) diverse and representative training datasets; (4) clear regulatory pathways and reimbursement frameworks; and (5) interdisciplinary collaboration among clinicians, data scientists, ethicists, and policymakers.

With these foundations, AI has the potential to shift atherosclerosis care from a reactive model to one that is more predictive, preventive, and personalized. However, this transition must be evidence-driven, patient-centered, and ethically grounded to ensure that AI benefits all populations equitably.

## Figures and Tables

**Table 1:** Comparative Overview of Traditional vs. AI-Based Risk Stratification in Atherosclerosis.

Aspect	Traditional Risk Models	AI/ML-Based Models	Clinical Implications
Core Methodology	Linear regression; assumes additive relationships	Non-linear algorithms (RF, SVM, GBM, NN); captures complex interactions	Traditional models may miss synergistic risk; AI better reflects biological complexity
Data Sources	Limited clinical variables (age, sex, BP, lipids, smoking, diabetes)	High-dimensional: genomics, proteomics, imaging, wearables, lifestyle, social determinants	AI enables precision risk assessment but requires robust data infrastructure
Temporal Dynamics	Static, single-time-point assessment	Continuous learning from time-series and updated data	AI supports dynamic, adaptive risk stratification; requires ongoing model maintenance
Subgroup Discovery	Not applicable	Unsupervised phenotyping identifies hidden risk clusters	AI can detect high-risk patients missed by standard tools (e.g., young patients with elevated inflammatory biomarkers)
Clinical Validation	Extensive population-level validation	Variable; many models lack external validation	Urgent need for prospective, multi-center validation across diverse populations
Interpretability	High (transparent equations)	Variable; often “black box”	XAI techniques (SHAP, LIME) are critical for clinical trust and regulatory approval

**Table 2:** Application of AI in image analysis and plaque characterization in atherosclerosis.

Aspect	AI Applications in Imaging	Clinical Implementation	Limitations
Plaque Characterization Basis	Quantification of plaque burden (% stenosis, total volume); differentiation of plaque composition (calcified, fibrous, lipid-rich); detection of vulnerability markers (low-attenuation plaque, napkin-ring sign, thin-cap fibroatheroma, positive remodeling).	Automated plaque scoring in CCTA; calcium scoring widely available; vulnerability markers increasingly incorporated into advanced imaging workflows.	Limited standardization of vulnerability criteria; variability in imaging protocols; limited availability of annotated datasets across modalities.
Data Input Types	CCTA, CAC scoring, IVUS, OCT, MRI, radiomics-derived texture/shape features.	CCTA-based AI platforms (e.g., HeartFlow, Cleerly) entering clinical practice; automated CAC scoring embedded in some CT workstations.	High costs and limited access to OCT/IVUS outside tertiary centers; lack of interoperability between imaging vendors; radiomics still largely research-only.
AI Methods	CNNs for segmentation and classification; DL for plaque detection/quantification; radiomics/ML for vulnerability prediction; hybrid models combining imaging features with outcomes.	Automated CCTA plaque quantification is commercially available; hybrid AI-imaging tools are under evaluation in clinical trials.	Models often “black box” with limited explainability; need for external validation across diverse populations; and data privacy concerns.
Clinical Applications	Automated detection/segmentation of plaques; reproducible plaque burden and composition quantification; prediction of high-risk plaques; triage for invasive angiography; prognostic modeling linking plaque features to outcomes.	Risk stratification refinement in statin-treated patients; support for treatment decisions (e.g., intensifying lipid-lowering or anti-inflammatory therapy); improved efficiency in radiology/cardiology workflows.	Clinical adoption remains slow; reimbursement models unclear; integration into electronic health record (EHR) systems limited.
Advantages	Reduced inter-observer variability; high-throughput analysis; early identification of vulnerable plaques; extraction of subtle radiomic features invisible to human readers; reproducibility across centers.	Support for population-level screening and personalized interventions; potential to reduce missed high-risk plaques in routine imaging.	Over-reliance on AI outputs without clinical oversight; possible algorithmic bias from training data.
Limitations	Need for large annotated datasets; variability in imaging quality; lack of universal standardization; regulatory validation ongoing.	Current use mainly in specialized centers and research trials; gradual movement toward guideline integration.	Regulatory hurdles, medico-legal liability issues, and clinician skepticism slow widespread deployment.

**Table 3:** Clinical Readiness of AI Imaging Tools in Atherosclerosis.

Application	Regulatory Status	Clinical Implementation	Evidence Base
Automated CAC scoring	FDA-cleared; widely available	Routine clinical use in many centers	Extensive population-level validation
CCTA plaque quantification (volume, stenosis)	FDA cleared (multiple platforms)	Increasing adoption in advanced centers	Validated against IVUS/OCT; prospective outcome data emerging
FFRCT	FDA cleared; CPT coded; CMS coverage	Established clinical use	Multiple prospective trials
Plaque composition analysis (calcified vs. non-calcified)	Some platforms FDA cleared	Research and selected clinical use	Validated against invasive reference standards
Radiomics-based vulnerability prediction	Investigational	Research only	Retrospective cohort studies; prospective validation lacking
AI for plaque progression prediction	Investigational	Research only	Limited longitudinal data
AI-guided anti-inflammatory therapy selection	Investigational	Not in clinical use	Preclinical and early retrospective studies

**Table 4:** Ethical issues and considerations regarding the use of AI for the treatment of atherosclerosis.

Ethical Issue	Description	Implications	Key Considerations/Solutions
Algorithmic Bias	Training data may underrepresent certain populations (e.g., ethnic, gender, socioeconomic groups).	Risk of underdiagnosis or undertreatment in marginalized populations.	Use diverse datasets; validate models across subgroups to ensure fairness.
Data Privacy & Consent	AI depends on large volumes of sensitive health data from various sources.	Potential misuse or unauthorized access to personal health information.	Ensure transparent consent, opt-out mechanisms, and compliance with data laws.
Transparency & Explainability	Deep learning models may act as “black boxes” with unclear decision logic.	Undermines patient trust and clinician’s ability to interpret AI decisions.	Develop explainable AI (XAI); support shared decision-making with interpretable outputs.
Accountability & Liability	Ambiguity over responsibility for AI-related errors.	Legal and ethical uncertainty about who is liable when harm occurs.	Establish clear frameworks for clinical and legal responsibility.
Dehumanization of Care	Overreliance on AI may reduce clinician–patient interaction.	Loss of empathy, communication, and personalized care.	Position AI as a supportive tool, not a replacement for human judgment.
Equity & Access	Advanced AI systems may be inaccessible in low-resource settings.	Risk of widening global and socioeconomic health disparities.	Promote equitable AI deployment, training, and infrastructure support.

## Data Availability

No new data were created or analyzed in this study. Data sharing is not applicable to this article.
